# The utility of a structured mentorship program for enhancing competencies in global health

**DOI:** 10.7189/jogh.10.010301

**Published:** 2020-06

**Authors:** Michelle M Amri, Emily Kocsis, Shweta Dhawan, Dilani Logan, Christina Angelakis, Donald C Cole

**Affiliations:** 1 Dalla Lana School of Public Health, University of Toronto, Toronto, Canada; 2 Canadian Coalition for Global Health Research, Ottawa, Canada; 3 Faculty of Medicine, Dalhousie University, Halifax, Canada; 4 American Institutes for Research, Chapel Hill, North Carolina, USA; 5 Schulich School of Medicine & Dentistry, Western University, London, Canada

## THE NEED FOR FORMALIZED GLOBAL HEALTH MENTORSHIP

In an ever more globalized world, crises such as climate change, antimicrobial resistance, and income inequality, are some of the greatest challenges of our time. As a prominent example of the latter, the final report of the World Health Organization (WHO) Commission on the Social Determinants of Health, *Closing the Gap in a Generation*, synthesized knowledge on the causes of causes of health inequities, emphasizing the link between poor health and the “inequitable distribution of power, money, and resources’”[1]. To solve these seemingly intractable problems, a workforce that is adaptive, creative, and capable of working across disciplines is required. As a relatively new and expanding field, a comprehensive and collaborative approach to global health training and education is urgently needed [2].

While there remains little consensus on the most effective approaches for preparing students and young professionals (SYPs) to work in this expansive field, changing institutional and individual mindsets has been regarded as a key strategy [3]. Although informal mentorship relationships between experienced professionals and trainees certainly exist, formal, structured offerings are far less common [4]. Mentorship programs are instrumental for equipping students and young professionals with the proper competencies, such as critical thinking, creative problem solving, and effective verbal and communication skills to work in the challenging and cross-disciplinary field of global health. Consonant with the Beyond Flexner movement emphasis on the social mission of medicine in the United States, a global independent commission on education of health professionals in the 21^st^ century argued for training to address health disparities, with mentorship as an explicit mechanism to foster learning [5].

Established mentorship programs offer numerous professional and personal benefits to both trainees and mentors [6]. For example, mentors may aid young professionals in navigating uncertain and unclear career pathways, which is particularly needed in the cross-disciplinary field of health where one can transition to different roles [7]. By providing mentees the opportunity to speak with a variety of established professionals with diverse career paths, mentorship programs allow students to explore various career pathways and innumerable trajectories and roles available to those wishing to work in the field of global health (ie, career guidance and career choice) [6]. Moreover, mentorship within health has also been shown to play an instrumental role in promoting: opportunities for learning, employment performance, and multidisciplinary collaboration [6]. Further, mentors may aid SYPs in gaining valuable insights regarding their desired field of work, enhance their abilities to achieve their career goals, and facilitate connections between their peers and their SYP, providing networking opportunities to acquire a professional network, leading to career advancement [6].

## THE MENTORNET PROGRAM

Recognizing the insufficient presence of formalized mentorship programs, and the need for networking opportunities in the field of global health, a group of four students in Canada developed “MentorNet”, a structured global health mentorship program. With support from the Canadian Society of International Health (CSIH), the program was launched in 2011. The goals of the program were to provide SYPs from different disciplines with opportunities to learn and explore their interests in global health through a structured program with a mentor in the field.

Currently in its eighth year, MentorNet uses a module-based curriculum to support knowledge exchange, conversations, and reflections between SYP-mentor pairs. Each module consists of a two- to three-page document that introduces a global health topic, provides resources for learning and presents discussion questions to facilitate conversation between pairs. In addition to the modules and one-on-one interaction with their mentors, participants are also provided with an opportunity to network with other program participants through virtual mediums such as a Facebook group, global health conferences, and informal meet-ups.

Participation in the program spans the course of eight months starting at the beginning of the calendar year. As of 2017, MentorNet became open to international applicants from diverse backgrounds and receives over 150 SYP applications for 30-35 spots in the program. Selection is based on responses to an online application that evaluates interests and experiences in global health, demonstrated understanding of issues in global health, a clear indication of how mentorship would help advance their career, and flexibility to engage in program activities over eight months. Similarly, mentors are recruited through an online application process that evaluates experience in mentorship, background in global health, interest in the program, and availability during the program year. The responses are scored based on motivation to participate in mentorship, global health interest/experience, degree of professionalism, and overall effort invested in the application. After the highest scoring SYPs and mentors are identified, they are matched based on goals of participation in the program, area of interest within global health, and location (in that order). The educational backgrounds of SYPs applying to the program range from undergraduate to postgraduate in diverse disciplines such as health sciences, social sciences, and medicine. The pool of mentors complements this diversity with backgrounds in academia, medicine, clinical sciences, policy, management, and various other disciplines.

At the onset of the program, the SYP and mentor are introduced to each other, establish their learning objectives, and determine a mutually agreed upon frequency of communication. While most pairs choose to work on one module every month, the program is flexible in accommodating pairs who want to do more or fewer modules depending on their schedule. A steering committee member acts as a program liaison who works one-on-one with pairs to ensure that a clear understanding on the timing and frequency of communication is established, and to ensure that the pair achieves their learning goals. The pairs are sent resources via email on a monthly basis to facilitate conversation and encourage discourse by their program liaisons. Program liaisons act as points-of-contact for fostering an environment of openness which keeps the mentoring relationships moving forward.

## REFLECTIONS ON MENTORNET

On an individual level, MentorNet provides SYPs with formal and informal opportunities for professional development, networking, learning, and personal growth within the field of global health. By connecting the current and future global health workforce using pre-selected topics grounded in core global health issues and utilizing them to find common ground between mentors and SYPs, deeper professional relationships are built. The establishment of a formal mentorship program within the field of global health has generally improved SYPs understanding of global health issues, while also allowing them to foster more contacts and gain additional information about career opportunities, contributing to career guidance, choice, and management [6]. MentorNet enables mentor/SYP pairs to select the topics they are most interested in among an expansive list of modules during the first session. The modules help to build more intimate professional relationships and conversations about solutions to some key global health challenges through a thorough process of reflection. SYPs are also encouraged to provide recommendations for new modules, which allows for the expansion of global health topics. This in turn, can help stimulate ideas on key global health challenges, engaging both SYPs and established professionals in a collaborative discussion, including both open dialogue and critical thinking.

**Figure:**
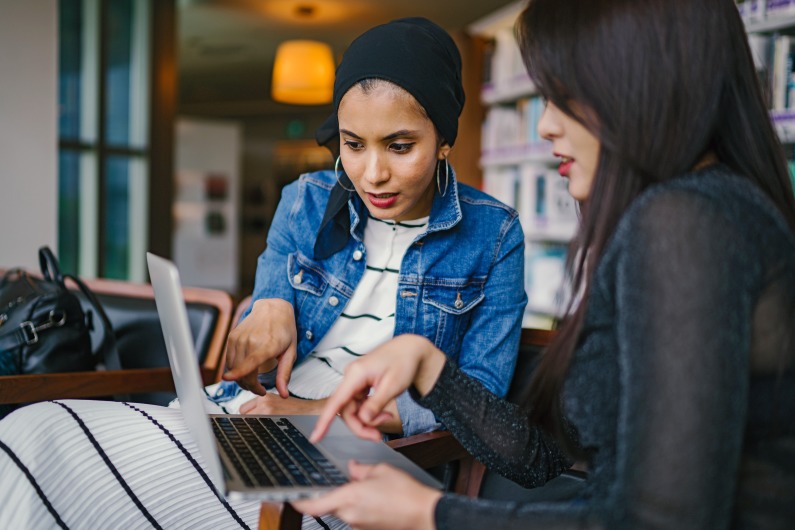
Photo: Two students and young professionals engaging in discussion while pointing to a laptop (by mentatdgt, used with permission from Pexels.com).

Furthermore, mentors inform SYPs about written and unwritten norms within the field of global health and allow SYPs to gain access to a broader social network channels (ie, “social capital”) [8]. The mentorship relationships formed over the course of the program have the potential to extend further and become highly-valued career-long relationships. The one-to-one connections that are formed have the potential to aid SYPs and mentors at varying points across their careers. For instance, SYPs are faced with a challenging transition period from academic to professional environments during which professional identity shifts and job ambiguity may increase. Individuals who have developed communication and problem-solving skills alongside knowledge of various perspectives, such as those fostered through mentorship programs, may be better equipped to deal with conflicting information revolving around professional responsibilities [9].

On a broader level, the structure of the MentorNet program provides an effective approach by which to educate students and younger professionals. When expanded upon, this could contribute to greater health system strengthening and workforce capacity building. As has been previously pointed out by other scholars, global health expertise is expansive and includes, but is not limited to, a plethora of topics including global governance, cultural sensitivity, global disease trends, and global development [10,11]. Mentorship is particularly important in this domain, as it might guide and teach SYPs about fundamental values in the field that are not communicated and taught in classroom settings [9]. Wherefore, in other fields the absence of mentorship has been attributed to feeling “out of the loop”, such as for females in law [8].

## CONCLUSION

MentorNet provides a strong example of how, when done thoughtfully, mentorship programs offer a relatively low-cost model by which SYPs can enter the field of global health with greater knowledge, connections, and awareness of professional opportunities. As a volunteer-run, primarily virtual program, the program carries very few costs, other than those associated with marketing and promotion.

The absence of a concrete professional identity emphasizes the importance of programs like MentorNet. Other global health associations may be able to adapt the MentorNet model to both meet SYPs needs and build their associations’ contribution to global health. Due to the vast interdisciplinary nature of global health, SYPs often require clarity and direction regarding all the possible avenues available to an individual wishing to pursue a career in global health. Ultimately, global health mentorship enables participants to have a low-cost and easily implementable opportunity to enhance their capacity through relationship building, research, and practice, while offering mentors with a chance to be introduced to innovative ideas and perspectives.
